# Management of dyslipidaemia in patients with chronic kidney disease: a position paper endorsed by the Italian Society of Nephrology

**DOI:** 10.1007/s40620-020-00707-2

**Published:** 2020-02-17

**Authors:** Roberto Pontremoli, Vincenzo Bellizzi, Stefano Bianchi, Roberto Bigazzi, Valeria Cernaro, Lucia Del Vecchio, Luca De Nicola, Giovanna Leoncini, Francesca Mallamaci, Carmine Zoccali, Michele Buemi

**Affiliations:** 1 Università degli Studi and I.R.C.C.S. Ospedale Policlinico San Martino, Viale Benedetto XV 6, 16132 Genoa, Italy; 2 Division of Nephrology, Dialysis and Transplantation, University Hospital “San Giovanni di Dio e Ruggi d’Aragona”, Via San Leonardo, 84131 Salerno, Italy; 3 Nephrology and Dialysis Complex Operative Unit, Department of Internal Medicine, ASL Toscana Nordovest, Livorno, Italy; 4 Unit of Nephrology and Dialysis, Department of Clinical and Experimental Medicine, University of Messina, Via Consolare Valeria 1, 98125 Messina, Italy; 5 Department of Nephrology and Dialysis, A. Manzoni Hospital, ASST Lecco, Lecco, Italy; 6 Nephrology Division, Department of Advanced Medical and Surgical Sciences, University of Campania “L. Vanvitelli”, Piazza Miraglia, 80138 Naples, Italy; 7 Nephrology, Dialysis and Transplantation Unit, Ospedali Riuniti, Reggio Calabria, Italy; 8 CNR-IFC, Clinical Epidemiology and Pathophysiology of Renal Diseases and Hypertension, Nefrologia-Ospedali Riuniti, 89100 Reggio Calabria, Italy

**Keywords:** Cardiovascular risk, Chronic kidney disease, Dyslipidaemia, Lipid lowering treatment, Statin

## Abstract

Chronic kidney disease (CKD) represents a major public health issue worldwide and entails a high burden of cardiovascular events and mortality. Dyslipidaemia is common in patients with CKD and it is characterized by a highly atherogenic profile with relatively low levels of HDL-cholesterol and high levels of triglyceride and oxidized LDL-cholesterol. Overall, current literature indicates that lowering LDL-cholesterol is beneficial for preventing major atherosclerotic events in patients with CKD and in kidney transplant recipients while the evidence is less clear in patients on dialysis. Lipid lowering treatment is recommended in all patients with stage 3 CKD or worse, independently of baseline LDL-cholesterol levels. Statin and ezetimibe are the cornerstones in the management of dyslipidaemia in patients with CKD, however alternative and emerging lipid-lowering therapies may acquire a central role in near future. This position paper endorsed by the Italian Society of Nephrology aims at providing useful information on the topic of dyslipidaemia in CKD and at assisting decision making in the management of these patients.

## Introduction

Chronic Kidney Disease (CKD) is considered a major public health issue. On a worldwide scale the prevalence of this condition is about 10% [1–3]. CKD entails a high economic cost to health systems since it is an independent risk factor for cardiovascular (CV) disease and a risk multiplier. The majority of patients with CKD are more likely to die prematurely due to CV disease than to survive long enough to reach end-stage kidney disease (ESKD) [4]. The relationship between reduction of glomerular filtration rate (GFR) and/or increase of albuminuria and CV risk is graded and holds true even in the setting of early renal impairment [5, 6]. As kidney function declines, kidney specific risk factors play a progressively increasing role in the high CV and renal risk of CKD patients [7]. On the other hand, traditional, modifiable risk factors like hypertension, diabetes and dyslipidaemia contribute to the high risk of premature CV events in CKD in stages 1–4 [8]. Therefore, interventions to modify these risk factors are of paramount importance for CV prevention in this population.

## Dyslipidaemia as a cardiovascular and renal risk factor in CKD

Alterations of the lipid profile are common in patients with all stages of CKD, in dialysis and renal transplant patients. Patients with advanced CKD or ESKD show a characteristic lipid pattern of hypertriglyceridemia and low high-density lipoprotein (HDL)-cholesterol levels but normal low-density lipoprotein (LDL)-cholesterol concentrations. In patients with ESKD, LDL-cholesterol shows a negative relationship with mortality at below average LDL-cholesterol levels and a flat or weakly positive relationship at high LDL-cholesterol levels [9, 10]. A large wealth of data supports the notion that dyslipidaemia through atherosclerosis contributes to the high CV morbidity and mortality of CKD patients (including dialysis and renal transplantation).

## Abnormalities of lipid profile in CKD

At the end of 90 s, Kasiske evaluated the abnormalities of lipid profile in CKD by pooling data from several studies [11]. In this systematic review, it emerged that dyslipidaemia is a common problem in CKD patients and that alterations in the lipid profile in this population differ from those typically seen in non-CKD individuals. Some heterogeneity in the lipid profile can be observed also within the CKD population depending on the presence of renal damage (proteinuria), non-traditional risk factors (e.g. systemic inflammation), type of renal replacement treatment (peritoneal vs extracorporeal dialysis) and the use of immunosuppressive agents (in kidney transplant).

In 2008 an in-depth analysis based on the Multi-Ethnic Study of Atherosclerosis (MESA), a cohort study that enrolled 5109 participants with early CKD [estimated GFR (eGFR) range 90–60 ml/min/1.73 m^2^] [12], showed that even a mild decrease of eGFR associates with higher levels of triglycerides and with a reduction in HDL serum concentration while LDL concentration measured by conventional methods was unaltered. However, when LDL subclasses were examined by nuclear magnetic resonance, the concentrations of atherogenic small LDL particles and intermediate-density particles (IDL) were raised in CKD patients.

Thus, lipoprotein abnormalities start early in the course of CKD, differ from the typical pattern in general population, and are also heterogeneous within the CKD population (Table 1).

**Table 1:** Abnormalities of lipid profile by target population (modified from ref [9, 10])

	Nephrotic syndrome	CKD (stages 1–2)	CKD (stages 3–4)	HD	PD	KTR
Total Cholesterol	↑↑	=	=	= or ↓	↑	↑
LDL	↑↑	=	= or ↓	= or ↓	↑	↑
HDL	↓	↓	↓	↓	↓	= or ↓
Triglycerides	↑↑	↑↑	↑↑	= or ↑	↑↑	↑ or ↑↑

*HD* haemodialysis, *HDL* high-density lipoprotein cholesterol, *KTR* kidney transplant recipient, *LDL* low-density lipoprotein cholesterol, *PD* peritoneal dialysis

As shown in Table 1, a common feature of CKD-related dyslipidaemia is the dysregulation of triglycerides and HDL. Experimental studies have shown that the significant increase in serum triglycerides depends on the impaired clearance of triglyceride-rich lipoproteins and their atherogenic remnants, while impaired maturation of HDL is mainly due to downregulation of lecithin-cholesterol acyltransferase (LCAT) and, to a lesser extent, to increased plasma cholesteryl ester transfer protein (CETP) [13]. Interestingly, also the HDL-mediated reverse cholesterol transport (i.e., the disposal of surplus cholesterol from peripheral tissues) and the HDL antioxidant and anti-inflammatory activity are impaired in CKD, particularly so in ESKD [14]. Similar to HDL, also LDL particles are qualitatively modified by oxidation thus becoming more atherogenic as compared to non-oxidized LDL particles [15].

Finally, high levels of “remnant cholesterol”, that is the cholesterol carried by non-HDL and non-LDL particles which includes chylomicrons, very low-density lipoproteins (VLDL) and IDL most likely represent an important pro-atherogenic risk factor in CKD [16].

**Table:** 

Consensus statement 1	
Global CV risk management is a priority in CKD patients at all stages. CKD is acknowledged as a coronary artery disease (CAD) risk equivalent and complete assessment of lipid status (including total and HDL-cholesterol, triglycerides and LDL estimation) is mandatory to devise optimal therapeutic strategy	

## Lipid lowering therapeutic strategies

### Diet and lifestyle modifications

Even though conclusive evidence is still lacking, there is wide agreement that a healthy lifestyle including a low content of saturated fats in the diet may reduce CV risk in the general population. The Mediterranean and the DASH diets are possible approaches to reduce the intake of saturated fats.

Experimental studies showed nephrotoxicity following a cholesterol-rich diet [17, 18]. Cross-sectional data associate a healthy diet with a high consumption of whole grain, fruits, vegetable and unsaturated fats with lower body mass index, serum LDL-cholesterol, total cholesterol, and fasting triglyceride concentrations across three major Asian ethnic groups [19]. Similarly, another cross-sectional study in Taiwan associated a frequent intake of fish and vegetables with a non-significant tendency to higher eGFR but not with the urine albumin/creatinine ratio [20]. To date, there are no dietary or pharmacological interventional studies demonstrating an amelioration of renal endpoints. A small, randomised trial of 40 stage-2 CKD patients showed an improvement in lipid parameters by the Mediterranean diet for 3 months but no GFR changes [21]. A low or very low protein diet may improve lipid parameters in CKD patients [22, 23] and a low fat diet may improve lipid profile in renal transplant recipients [24]. However, according to a meta-analysis of small trials, a very low protein diet supplemented with keto-analogues has no effect on lipid parameters in ESKD patients [25].

In CKD patients there is little evidence of the benefits of following healthy life-style including a diet with a low content of saturated fats. It is reasonable to hypothesize that the benefits observed in the general population may apply also in CKD patients (except for those on dialysis). The diet with a low content of saturated fats may also be part of other diets, such as the low-protein diet (including vegetarian and vegan ones) and the Mediterranean diet. Caution is needed in increasing the intake of fruits and vegetables in patients who are at risk of hyperkalaemia. Dietary interventions that may reduce serum triglycerides include a low-fat diet (15% total calories), reduction of monosaccharide and disaccharide intake and of total amount of dietary carbohydrates, and use of fish oils to replace long-chain triglycerides. Dietary modification should be used judiciously in individuals who are malnourished.

### Statins

The safety and efficacy of statins have been widely demonstrated both in primary prevention in patients at high CV risk [26, 27] and in secondary prevention after an atherosclerotic CV event [28, 29].

The presence of a reduced renal function represents, like diabetes mellitus or arterial hypertension, a significant CV risk factor [30] and secondary analyses of clinical trials showed that statin therapy may reduce the incidence of CV events in patients with CKD [31, 32]. The Table 2 summarises the effects of statins and the treatment indications for CKD patients.

**Table 2:** Statin therapy in patients with CKD

Target/mechanism of action	Inhibition of 3-hydroxy-3-methylglutaryl-coenzyme A (HMG-CoA) reductase		
Therapeutic target	LDL-cholesterol < 70 mg/dL in patients at high CV risk and < 55 mg/dL in patients at very high CV risk [55]		
Potential pleiotropic effects	Pro-inflammatory cytokines and CRP reduction, increased eNOS expression and activity, ROS reduction, atherosclerotic plaque stabilization, platelet aggregation inhibition, fibrosis and left ventricular hypertrophy decrease, reduction in migration and proliferation of vascular smooth muscle cells, nephroprotection, antitumor activity, immunomodulation [56, 57]		
Main adverse events	Myopathy (including myositis), rhabdomyolysis with or without AKI, myalgia, muscle cramps, asthenia, hepatitis/jaundice, increased blood levels of liver enzymes, alkaline phosphatase, CPK, HbA1c, and fasting glucose		
	CKD under conservative therapy	Haemodialysis and Peritoneal dialysis patients	Renal transplantation
Indications according to guidelines KDIGO [37] Joint British Societies for the prevention of CV disease [58] NICE [59] Canadian CV Society [60] ESC/EAS [55]	Estimation of CV risk not required if GFR < 60 ml/min/1.73 m^2^ or if albuminuria is present Indications: Adults ≥ 50 yrs with GFR < 60 ml/min/1.73 m^2^: statin or statin/ezetimibe therapy recommended Adults ≥ 50 yrs with CKD and GFR ≥ 60 ml/min/1.73 m^2^: statin therapy recommended Adults 18–49 yrs: statin therapy suggested in the presence of one or more of the following: DM, known coronary disease, estimated 10-year incidence of non-fatal MI or coronary death > 10%, previous ischaemic stroke	It is suggested not to start statin or statin/ezetimi-be therapy It is suggested to continue treatment in patients already on statin or statin/ezetimi-be therapy at the time of dialysis initiation	Statin therapy suggested
Molecules and dosages resulted effective in clinical studies	Lovastatin (20 mg/day) [61]		Lovastatin (20 mg/day) [81–83]
	Pravastatin (10–20 mg/day) [62–65]		Pravastatin (20 mg/day) [84–86]
	Atorvastatin (10–80 mg/day) [32, 66–71]		Atorvastatin (10 mg/day)[87, 88]
	Simvastatin (10–40 mg/day) [72–76]		Simvastatin (5–40 mg/day) [81, 85, 89–95] or Simvastatin-Ezetimibe (extrapolated from the SHARP trial)
	Simvastatin-Ezetimibe (SHARP trial) [33]		Fluvastatin (20–80 mg/day) [51, 82, 96–98]
	Fluvastatin (10–30 mg/day) [77]		Rosuvastatin (10 mg/day) [99]
	Rosuvastatin (2.5–20 mg/day) [78–80]		

*AKI* acute kidney injury, *CKD* chronic kidney disease, *CPK* creatine phosphokinase, *CRP* C-reactive protein, *CV* cardiovascular, *DM* diabetes mellitus, *eNOS* endothelial nitric oxide synthase, *GFR* glomerular filtration rate, *HbA1c* glycated haemoglobin, *HDL* high-density lipoprotein, *LDL* low-density lipoprotein, *MI* myocardial infarction, *ROS* reactive oxygen species, *yrs* years

Administration of statins (generally at doses equivalent to 20 mg of simvastatin) has been shown to reduce CV and all-cause mortality and prevent major CV events in stage 1–4 CKD patients. The landmark study of heart and renal protection (SHARP), in a cohort of 9270 stage 3–5 CKD patients including 1533 patients on dialysis (83% on haemodialysis) randomised to a simvastatin plus ezetimibe or placebo treatment, showed that lipid lowering intervention reduces the risk of a combined endpoint including non-fatal myocardial infarction or coronary death, non-haemorrhagic stroke, or any arterial revascularisation procedure by the 17% [33]. However, a meta-analysis including dialysis patients enrolled in SHARP as well as previous major statin-based trials in these patients like the 4D [34] and AURORA [35] trials, showed no clear benefit of statins in this population [36]. Based on this evidence, the KDIGO guidelines advise not to start statin therapy in dialysis patients but to continue it if previously established [37].

Several trials of small dimension tested the effect of statins in ESKD patients with dyslipidaemia undergoing peritoneal dialysis [38–49]. In these trials statins reduced serum total cholesterol, LDL-cholesterol, triglycerides, apolipoprotein B as well as markers of inflammation and endothelial dysfunction, and increased HDL-cholesterol and apoprotein A1 concentrations compared to placebo. In general, statin administration was well tolerated. However, there is absolutely no evidence of benefits of statins on major clinical endpoints such as mortality or CV events in this population.

As for kidney transplant patients, the latest Cochrane meta-analysis (22 studies, 3465 participants), published in 2014 [50], showed that statins administered at a dose equivalent to simvastatin 10 mg/day can reduce CV events, although the effects of treatment on outcomes such as overall mortality, stroke, renal function and toxicity remain uncertain. Most of the data pooled in this meta-analysis were from the ALERT 2001 study [51], which provided about 2/3 of patients included in the meta-analysis.

Overall, therapy with statins is recommended in pre-dialysis CKD patients and possibly also in renal transplant recipients, whereas initiation of treatment is not recommended in haemodialysis or in peritoneal dialysis patients. However, in patients already on statin or statin/ezetimibe therapy at the time of dialysis initiation continuation of treatment should be considered especially in the presence of atherosclerotic vascular disease.

Despite preliminary favourable data [52, 53], there seems to be no meaningful effects of statins on the progression of CKD [54].

### Ezetimibe

Ezetimibe (1-(4-fluorophenyl)-(3R)-[3-(4-fluorophenyl)-(3S)-hydroxypropyl]-(4S)-(4-hydroxyphenyl)-2-azetidinone) [100] inhibits cholesterol and phytosterol intestinal absorption and reduces plasma cholesterol by 15–20% in humans. The target of ezetimibe is the NPC1L1 (Niemann-Pick C1-Like 1) transporter, which is localized on the enterocyte brush border and plays a key role in the trans-membrane transport of cholesterol in the small intestine [101, 102]. The Table 3 describes the effects of ezetimibe and the treatment indications for patients with renal disease.

**Table 3:** Ezetimibe therapy in patients with CKD

EZETIMIBE				
Target/mechanism of action	Selective inhibition of NPC1L1 protein, transporter of food cholesterol from intestinal lumen into enterocytes [101, 102]			
Therapeutic targets in CKD	LDL-cholesterol < 70 mg/dL in patients at high CV risk and < 55 mg/dL in patients at very high CV risk [55]			
Potential pleiotropic effects	Enhancement of plaque regression through: inhibition of intestinal absorption of plant sterols (associated with early atherosclerosis in some studies); modulation of genes involved in inflammation and/or oxidative stress, inhibition of the differentiation of monocytes and/or macrophages, inhibition of proliferation of smooth muscle cells; inhibition of platelet aggregation and activation; modulation of atherosclerotic plaque composition [117–123]			
Main adverse effects	Asthenia, myalgia, arthralgia, increased levels of liver enzymes and creatine phosphokinase, diarrhoea, dyspepsia, gastritis, headache			
	CKD on conservative therapy	Extracorporeal dialysis	Peritoneal dialysis	Renal transplantation
Dosages tested in clinical studies and indications according to guidelines KDIGO [37] Joint British Societies for the prevention of CV disease [56] NICE [57] Canadian CV Society [58] ESC/EAS [55]	Ezetimibe monotherapy not recommended Adults ≥ 50 yrs with GFR < 60 ml/min/1.73 m2: statin or statin/ezetimibe therapy recommended	It is suggested not to start statin or statin/ezetimi-be therapy It is suggested to continue treatment in patients already on statin or statin/ezetimi-be therapy at the time of dialysis initiation	Ezetimibe 10 mg + simvastatin 10 or 20 mg/day [33]	No specific indication for ezetimibe therapy

*CKD* chronic kidney disease, *CV* cardiovascular, *LDL* low-density lipoprotein, *NPC1L1* Niemann-Pick C1-Like 1

According to KDIGO guidelines [37], ezetimibe monotherapy is not recommended in CKD patients since there is only scarce evidence of its effectiveness on relevant clinical outcomes [103, 104]. Conversely, mainly based on the results of the randomised double-blind SHARP (Study of Heart and Renal Protection) trial [33], the use of statin/ezetimibe association is formally recommended in predialysis CKD patients (see above and Table 2). This therapeutic approach allows avoiding the use of high statin doses with potentially reduced risk of myopathy and other adverse effects.

Very few studies enrolling a small number of patients have been performed to evaluate the safety and efficacy of the ezetimibe/statin association in peritoneal dialysis. These data suggest that such an association may induce an additional decrease in LDL levels allowing a reduction in the statin dose and related side effects [105].

Ezetimibe has also been shown to be effective in small cohorts of kidney transplant recipients with hypercholesterolemia in some, mostly not placebo-controlled, clinical studies [106–115], especially if associated with a statin. Hence, evidence is low and current guidelines suggest statin therapy with no specific indication for ezetimibe in transplanted patients. Moreover, the potential interaction with immunosuppressants deserves careful monitoring in order to prevent allograft dysfunction [116].

### Fibrates

The analogues of fibric acid (i.e. gemfibrozil, fenofibrate, bezafibrate, ciprofibrate) impact on the lipoprotein and triglyceride synthesis in the liver and their final major effect is the reduction of serum triglyceride levels. A secondary effect of these drugs is a small increase in HDL. Overall, fibrates reduce serum triglycerides in patients with hypertriglyceridemia and can be also used in association with other lipid-lowering agents to increase HDL-cholesterol. In general, these drugs are fairly well tolerated but may induce mild gastrointestinal, skin, liver and muscle symptoms.

During fibrate treatment, there may be an increase in serum creatinine particularly in elderly people, in those with pre-existing renal dysfunction and when these drugs are used at high-doses or in combination with renin–angiotensin–aldosterone (RAAS)-inhibitors. However, the creatinine rise by fibrates most often is transient and reversible (Table 4). A meta-analysis of randomised controlled trials comparing fibrates vs placebo in CKD patients concluded that fibrates improve lipid profiles and prevent CV events but the effects on kidney outcomes remain unknown [124]. A population-based study showed an increase in serum creatinine and a small increase in hospitalisations in elderly patients starting fibrates; no effect on acute kidney injury or mortality, however, was observed [125]. Within the frame of the Fenofibrate Intervention and Event Lowering in Diabetes (FIELD) trial in type 2 diabetics, a sub-analysis focusing on patients with stage 3 CKD (eGFR 60–30 ml/min/1.73 m^2^) showed a risk reduction (− 32%) in these patients of the same order of that in those with eGFR > 90 ml/min/1.73 m^2^ (− 15%) (P for interaction 0.2) [126] with no safety concern. In contrast, in an extended follow-up (10 years) analysis of the Action to Control Cardiovascular Risk in Diabetes (ACCORD) trial, a multifactorial intervention study in type 2 diabetics at high risk for CV disease, fenofibrate added to simvastatin doubled the risk for creatinine doubling [127] suggesting that fenofibrate may actually increase the risk for adverse kidney events.

**Table 4:** Fibrate therapy in patients with CKD

FIBRATES	
Target/mechanism of action	Fibrates interact with the liver peroxisome proliferator activated receptors (PPARs isotype-α), increasing the lipoprotein lipase activity and decreasing the synthesis and serum levels of triglycerides
Targets in other diseases	Reduction of triglycerides by 35–50%, increase in HDL-cholesterol by 5 (mono-therapy) to 20% (patients with triglycerides > 500 mg/dl)
Potential pleiotropic effects	The fibric acid derivatives may lower serum fibrinogen levels. Fenofibrate reduces serum uric acid levels.
Main adverse effects	Fairly well tolerated. Rare gastrointestinal symptoms, urticaria, myalgias (mainly in combination with statins); transaminases and alkaline phosphatase minor increase; increase in bile lithogenicity Fibrates have been linked to creatinine increase but these changes may be related to a class effect of other PPARs isotype- α agonists
Drug interaction	Possible enhancement of oral anticoagulant effects (warfarin) Fenofibrate may increase the clearance of cyclosporine and reduces serum cyclosporine levels (in heart transplant patients)
Recommendations	Fibrates may be second-line agents to reduce triglycerides in nephrotic syndrome The combination of fibrate and statin should be avoided (myositis) Bezafibrate is primarily excreted by the kidney and should be avoided in CKD A close monitoring of serum creatinine is mandatory during fenofibrate treatment, mainly in elderly, diabetic, high-risk patients and discontinuation of the drug should be suggested for a creatinine increase > 30%. Fenofibrate should be used with caution in renal transplant recipients on cyclosporine whose levels have to be regularly monitored Fibrates are contraindicated in patients with GFR < 15 mL/min/1.73 m^2^)

*CKD* chronic kidney disease, *HDL* high-density lipoprotein

In a small randomised trial with a crossover design in 11 patients with nephrotic syndrome gemfibrozil reduced serum triglycerides by the 51% and to a much lesser extent (− 13%) LDL-cholesterol as compared to placebo [128]. Fibric acid derivatives are not recommended to prevent pancreatitis or reduce CV risk in adults with CKD and hypertriglyceridemia.

### Bile acid sequestrants

The bile acid resins or sequestrants (first-generation: cholestyramine, colestipol; second-generation: colesevelam, colestimide) bind the bile acids in the intestine forming a non-absorbable complex, hence reducing their ability to solubilize lipids. Reduced reabsorption of bile acids stimulates the synthesis of these compounds in the liver which eventually results in lower LDL-cholesterol levels both in the bile and in plasma. Overall, the bile acid resins have low efficacy to reduce serum lipids and can be used as a sole lipid lowering treatment only in patients with mild to moderate elevations of LDL. Furthermore, they can be used in association with other lipid-lowering drugs in more severe hypercholesterolemia not associated with hypertriglyceridemia. In general, these drugs are safe but are scarcely used due to their gastrointestinal side effects [129–132].

No relevant research data exist on bile acid sequestrants in pre-dialysis CKD patients or in dialysis and renal transplanted patients (Table 5).

**Table 5:** Bile acid sequestrant therapy in patients with CKD

Bile acid sequestrants	
Target/mechanism of action	They bind the bile acids in the intestine, reducing the lipids solubilisation and the absorption of cholesterol. They reduce the reabsorption of bile acids, increasing their liver synthesis and reducing the LDL-cholesterol. The increase in bile-acid production causes the increase of triglyceride synthesis in the liver
Targets in other diseases	Reduction of LDL-cholesterol by 10–15% (25% at maximum dosage, but with low gastrointestinal tolerance). One report focusing on colestipol described a decrease of LDL-cholesterol up to 30% in patients with nephrotic syndrome
Main adverse effects	Bloating and constipation at the maximal dosage (low compliance). Increased serum triglyceride levels (transiently when triglycerides are at normal levels; contraindicated with triglycerides > 250 mg/dl). These drugs are chloride salts and hyperchloremic acidosis may be possible (rarely); volume depletion, renal failure and use of spironolactone may increase this risk
Drug interaction	These drugs may interfere with the absorption of many other drugs (i.e. thiazides, furosemide, propranolol, thyroxine, digoxin, warfarin, etc.); Colesevelam seems to interfere less than other drugs of the same class with the pharmacokinetics of other medications
Recommendations	In CKD every stage the bile acid sequestrants may be proposed as second-line agents in association with other lipid-lowering drugs in patients with incompletely controlled LDL-cholesterol levels and normal triglycerides It is recommended to assume other medications 1 h before or 3–4 h after bile acids sequestrants

*CKD* chronic kidney disease, *LDL* low-density lipoprotein

### Omega-3 fatty acids

Omega-3 polyunsaturated fatty acids, including docosahexaenoic acid (DHA) and eicosapentaenoic acid (EPA), effectively reduce triglyceride levels by 20–50% at optimal, recommended dosages. In vitro and in vivo experimental studies showing antiinflammatory, antioxidative as well as atherosclerotic plaque-stabilizing activity by omega-3 fatty acids suggest that these compounds may have CV and renal protective effects. However, the role of omega-3 fatty acid supplementation in the management of patients with CKD still remains unclear. Clinical research on these compounds initially focused on IgA nephropathy and was subsequently extended to other renal diseases, including patients with CKD. Clinical trials have shown conflicting results, probably related to different doses, EPA/DHA ratio, duration of therapy, and sample size of the study populations. Meta-analyses have been limited by the quality of available studies. To date, there is insufficient evidence to recommend the use of omega-3 fatty acids to prevent death, CV events or CKD progression both in pre-dialysis CKD and in ESKD patients [133–135]. The recent REDUCE-IT (Reduction of Cardiovascular Events with Icosapent Ethyl—Intervention Trial) supports a CV protective effect of EPA [136]. This multicentre, international trial included more than 8000 adults at high CV risk with well controlled LDL cholesterol on statins and with either established CV disease or diabetes mellitus and at least one additional CV risk factor. Furthermore, to be enrolled, patients in this trial had to have persistently elevated triglyceride levels (150–499 mg/dL). After a follow-up of 4.9 years, patients assigned to receive 4 g/day of highly purified, stable EPA had a relative risk reduction of 25% for a composite endpoint, including CV death, nonfatal myocardial infarction, nonfatal stroke, coronary revascularization, or unstable angina, as compared to placebo. Of note, the benefit observed with EPA was extended also to stage 3 CKD patients (eGFR 60–30 ml/min/1.73 m^2^). Further large-scale and long-term clinical trials including patients with stage 4 and 5 CKD are needed to confirm a role of EPA in CV prevention in CKD patients.

### PCSK-9 inhibitors

The proprotein convertase subtilisin/kexin type 9 (PCSK9) is a proteasis that induces the degradation of the LDL receptor. Evolocumab and Alirocumab are monoclonal antibodies targeting PCSK9 that effectively reduce serum cholesterol until median values of 20–30 mg/dL on top of maximised statin therapy. These drugs have been tested extensively in subjects with familiar hypercholesterolemia or at high CV risk and with LDL-cholesterol not at target despite statins or intolerant to statins [137].

The FOURIER (Further Cardiovascular Outcomes Research with PCSK9 Inhibition in Subjects with Elevated Risk) [138] and the ODYSSEY OUTCOMES [139] are the two largest trials testing this class of drugs. In the ODISSEY OUTCOMES a significant risk reduction for the combined primary CV endpoint (death from coronary heart disease, nonfatal myocardial infarction, fatal or nonfatal ischemic stroke, or unstable angina requiring hospitalization) (− 15%) and for death (− 15%) was observed in patients randomized to alirocumab (subcutaneous dose of 75 mg every 2 weeks) in comparison to those receiving placebo [139]. FOURIER, a trial testing evolocumab (140 mg every 2 weeks or 420 mg once per month) showed a risk reduction of the same magnitude (− 15%) with this drug for a composite endpoint including CV death, myocardial infarction, stroke, hospital admission for unstable angina, or coronary revascularization [138] and this was true also in patients with diabetes (− 17%) [140]. The benefit of these drugs seem higher in those who have higher baseline serum cholesterol [139] or in those at very high CV risk [141]. At present no significant safety concerns emerged and immunogenicity does not seem to be an issue for these two fully human antibodies. The number of patients to be treated to prevent CV events included in the combined endpoint of these trial is relatively elevated (nearly 70) and the cost of these agents is high. A recent post hoc analysis of ODISSEY OUTCOMES showed similar safety and efficacy in reducing serum LDL-cholesterol, lipoprotein (a), non-high-density lipoprotein cholesterol, apolipoprotein B, and triglycerides in patients with GFR of 30–59 ml/min/1.73 m^2^ as compared to those with higher GFR values [142]. Similarly greater CV protection with evolocumab as compared to placebo on top of intensive lipid-lowering treatment has been reported in a retrospective analysis of the FOURIER study in patients with stage 3 CKD [143]. Additional trials are needed to assess the efficacy of these drugs in patients with more severe degree of renal damage. No effect on renal progression has been observed by the use of PCSK-9 inhibitors.

**Table:** 

Consensus statement 2	
Therapeutic inertia is common in lipid management of CKD patients. LDL reduction should be considered as the primary target of therapy and pharmacologic intervention using statins with or without ezetimibe is recommended in all patients with stage 3–4 CKD irrespective of baseline values to achieve CV protection. The effect of lipid-lowering treatment on proteinuria and renal disease progression requires further evaluation.	

## Therapeutic targets of LDL-cholesterol and triglycerides

CKD is a condition of high CV risk, regardless of the presence of other comorbidities, and in patients with CKD LDL-cholesterol is a risk factor for atherosclerotic-related CV events [144].

In patients with CKD not on dialysis, LDL-cholesterol lowering therapy has shown to induce a significant reduction of CV events [145, 146].

The therapeutic targets of LDL-cholesterol to be achieved in patients with CKD do not differ substantially from those recommended in patients without CKD, with the exception of patients on dialysis [37, 55, 147]. In stage 3 CKD, according to the 2019 ESC/EAS Guidelines for the Management of Dyslipidaemias [55], LDL < 70 mg/dL and a reduction of at least 50% from “baseline”, are indicated, while in stage 4 and stage 5 non dialysis-dependent CKD patients (i.e. eGFR < 30 ml/min*1.73 m^2^) treatment goal should be LDL < 55 mg/dL, and a reduction of at least 50% from “baseline”.

The recommended therapeutic target can be achieved by the use of an adequate efficacy drug or drug combination or by progressive up-titration [55, 147]. Regardless of the modality of therapeutic approach, awareness of the importance of statin use in CKD should increase in tertiary nephrology care where clinical inertia on statin use still remains high [148].

Table 6 summarises the guidelines recommendations for LDL-cholesterol management among patients with CKD on conservative therapy, CKD on dialysis or renal transplant recipients.

**Table 6:** Guideline recommendations for LDL-cholesterol management in CKD patients

Guideline	Population	CKD stage	Treatment recommendations
KDIGO (2013)	Adults ≥ 50 years	1–2	Statin
		3–5 (not on dialysis)	Statin; Statin + Ezetimibe
	Adults 18–49 years + ≥ 1 of the following: 1. known coronary disease 2.DM 3. Prior ischemic stroke 4. estimated 10 years incidence of coronary death or non fatal MI > 10%	1–5 (not on dialysis)	Statin
	Adults with dialysis-dependent CKD	5 (HD or PD)	Statins or statin combinations should not be initiated; they can be continued if already received at the time of dialysis initiation
	Adult kidney transplant recipient	1–5	Statin
ACC/AHA (2018)	Adults with clinical ASCVD	1–5 (not on dialysis)	High-intensity statin preferred; moderate-intensity statin if not a candidate for high-intensity
	Adults with LDL-cholesterol ≥ 190 mg/dL	1–5 (not on dialysis)	High-intensity statin preferred; moderate-intensity statin if not a candidate for high-intensity
	Adults 40–75 years with DM and LDL-cholesterol 70–189 mg/dL (no ASCVD)	1–5 (not on dialysis)	High-intensity statin if estimated 10-years ASCVD risk ≥7.5%; moderate-intensity statin if not a candidate for high-intensity
	Adults 40–75 years with estimated 10-years ASCVD risk ≥ 7.5% (no DM or ASCVD)	1–5 (not on dialysis)	Moderate- or high-intensity statin
	Adults with dialysis-dependent CKD and kidney transplant recipients	5 (HD or PD) or 1–5 in kidney transplant recipient	No recommendation
ESC/EAS (2019)	Adults	1–5 (not on dialysis)	Statin; Statin + Ezetimibe
	Patients with dialysis-dependent CKD and free of atherosclerotic CVD	5 (HD or PD)	Statins should not be initiated; in patients already on treatment at the time of dialysis initiation, these drugs should be continued, particularly in patients with CVD
	Adult kidney transplant recipients	1–5	Treatment with statins may be considered

*ACC/AHA* American College of Cardiology/American Heart Association, *ASCVD* atherosclerotic cardiovascular disease, *CKD* chronic kidney disease, *CVD* cardiovascular disease, *DM* diabetes mellitus, *ESC/EAS* European Society of Cardiology/European Atherosclerosis Society, *HD* hemodialysis, *KDIGO* Kidney Disease Improving Global Outcomes, *LDL-cholesterol* low-density lipoprotein cholesterol, *MI* myocardial infarction, *PD* peritoneal dialysis

Guidelines on management of triglycerides in patients with CKD do not indicate specific target values, but suggest being reasonable to adopt therapeutic lifestyle changes (dietary modification, weight reduction, increased physical activity, decreased alcohol intake, and treatment of hyperglycaemia, if present) in patients with high fasting levels of serum triglycerides (> 5.65 mmol/l, > 500 mg/dL) [37]. Patients with triglyceride serum value higher than 200 mg/dL and high CV risk should be treated with a statin, regardless of the presence of CKD [37, 55].

**Table:** 

Consensus statement 3	
Patients with stage 3 CKD are considered to be at high risk and patients with stage 4–5 CKD or on dialysis at very high risk In the setting of non-dialysis CKD, goal of therapy is LDL < 70 mg/dL in high risk CKD patients and < 55 mg/dL in very high-risk patients.	

## Conclusions

CKD is a common condition, affecting around 10% of the adult population worldwide, and its prevalence rises sharply with increasing age. CKD entails a significant increase in CV morbidity and mortality. Dyslipidaemia, being one the most important modifiable risk factors associated to CKD, is a preferred target for treatment in order to reduce the CV risk burden. Several studies have shown that an optimal lipid control by means of statin with or without ezetimibe is associated with an improved CV outcome in CKD patients. Despite this evidence, therapeutic inertia is common in CKD patients. It is recommended that all patients with CKD stage 3 or worse receive lipid-lowering therapy to reach a LDL target value < 70 mg/dl irrespective of their baseline levels and/or clinical conditions preferably with a statin or statin/ezetimibe combination.
